# Myeloperoxidase promotes a tumorigenic microenvironment in non-small cell lung cancer

**DOI:** 10.1016/j.redox.2026.104399

**Published:** 2026-09-15

**Authors:** Paulina Valadez-Cosmes, Kathrin Maitz, Anna Lagler, Oliver Kindler, Nejra Cosic Mujkanovic, Sofia Raftopoulou, Melanie Kienzl, Zala Nikita Juvan, Ana Santiso, Luka Brcic, Gregor Gorkiewicz, Jörg Lindenmann, Wolfgang Sattler, Akos Heinemann, Rudolf Schicho, Gunther Marsche, A. McGarry Houghton, Julia Kargl

**Affiliations:** a Division of Pharmacology, Otto Loewi Research Center, Medical University of Graz, Graz, Austria; b BioTechMed, Graz, Austria; c Diagnostic and Research Institute of Pathology, Medical University of Graz, Graz, Austria; d Division of Thoracic and Hyperbaric Surgery, Department of Surgery, Medical University of Graz, Graz, Austria; e Division of Molecular Biology and Biochemistry, Gottfried Schatz Research Center, Medical University of Graz, Graz, Austria; f Clinical Research Division, Fred Hutchinson Cancer Research Center, Seattle, WA, United States; g Human Biology Division, Fred Hutchinson Cancer Research Center, Seattle, WA, United States; h Division of Pulmonary and Critical Care Medicine, University of Washington, Seattle, WA, United States

**Keywords:** Lung cancer, Myeloperoxidase, Neutrophils, T cells, CD8 T cells

## Abstract

Myeloperoxidase (MPO) is a heme peroxidase that is mainly expressed and secreted by neutrophils. MPO's role in inflammatory diseases has been highlighted in recent years, but its role in tumor development remains unclear. Therefore, we investigated the role of MPO in non-small cell lung cancer (NSCLC). *In silico* analysis revealed a survival benefit in patients with NSCLC and low MPO expression. Furthermore, a syngeneic tumor model using MPO knockout (KO) mice revealed that mice lacking MPO had lower tumor growth than controls. The reduction in tumor size was accompanied by an increase in lymphoid populations, including natural killer cells and CD8^+^ T cells, suggesting a shift to a more anti-tumorigenic immune environment in MPO-KO mouse tumors. The T cell induced interferon-gamma (IFN-γ) expression was increased in MPO-KO tumors, indicating increased tumoricidal activity. CD8 depletion abolished the previously observed reduction in tumor size in MPO-KO mice, indicating that CD8^+^ T cells play an important role. *In vitro,* T cells treated with MPO showed reduced proliferation and IFN-γ expression. Furthermore, MPO associated directly with T cells. Interestingly, MPO^+^ lymphocytes, including CD8^+^ T cells, were found in tumor samples from patients with NSCLC. Our findings suggest that MPO plays an immunosuppressive role in NSCLC.

## Teaser

**One Sentence Summary:** High myeloperoxidase (MPO) expression in non-small cell lung cancer patients is a predictor for adverse outcome and mice lacking MPO showed enhanced anti-tumorigenic leukocyte infiltration, suggesting a pro-tumorigenic role of MPO.

## 1 Introduction

Lung cancer remains the leading cause of cancer-related mortality worldwide [1]. Non-small cell lung cancer (NSCLC) accounts for 85% of all lung cancer diagnoses [2]. Despite recent advances in cancer therapy, the 5-year survival rate for patients with lung cancer remains dismal at around 15% [3]. The identification of novel therapeutic targets within the tumor microenvironment (TME) is crucial for developing new strategies to treat lung cancer.

Tumor-infiltrating neutrophils and CD8^+^ tumor-infiltrating lymphocytes are two important cell types that shape the TME. Despite their complexities, one of the main functions of CD8^+^ T cells within the TME is to recognize tumor-associated antigens and kill cancer cells [4]. Indeed, higher CD8^+^ T cell counts have been linked to better outcomes in breast [5], colorectal [6], and lung [7,8] cancers, among others. On the other hand, neutrophils have been reported to play ambivalent roles in cancer, including pro [[9], [10], [11]] and anti-tumorigenic [12,13] properties. Growing evidence suggests that tumor-infiltrating neutrophils can act as immunosuppressive cells and manipulate T cell behavior in favor of the tumor [14]. Infiltrating neutrophils are abundant in NSCLC [15]. Furthermore, some studies have linked neutrophil infiltration within tumors to poor clinical outcomes [16]. It has also been demonstrated that neutrophils suppress lymphocyte proliferation and function within the TME [17,18]. More recently, some neutrophil cytoplasmic granule components, including myeloperoxidase (MPO), have been proposed to contribute to tumor development [[19], [20], [21]].

MPO is a heme-containing enzyme that is primarily expressed by neutrophils [22] but is also found in small amounts in other myeloid-derived cells such as monocytes [23] and macrophages [24]. MPO is found in neutrophil primary (azurophilic) granules, accounting for 5% of total protein content [22]. MPO, like other peroxidases, catalyzes the production of many powerful oxidants, most notably the conversion of hydrogen peroxide and chloride ions to hypochlorous acid (HOCl) [25]. Upon neutrophil activation, MPO is secreted into the extracellular milieu during acute inflammation or in the TME, where it can cause tissue damage at inflammatory sites [26,27]. However, the role of MPO in tumor development remains poorly understood. In murine models of melanoma and pancreatic ductal adenocarcinoma, genetic deletion or inhibition of MPO has been shown to enhance the efficacy of immune checkpoint blockade, evidenced by reduced tumor growth [28,29]. In the pancreatic cancer model, MPO deficiency was also associated with a shift in the tumor microenvironment and diminished T cell suppression [29]. While these findings highlight MPO as a potential therapeutic target in cancer, data on its role in NSCLC are lacking, and the underlying mechanisms remain unclear.

The purpose of this study was to determine the role of MPO in the progression of lung cancer. In the present study, we show that MPO knockout (KO) mice had smaller tumors than wildtype (WT) littermates, indicating a pro-tumorigenic role for MPO in NSCLC. This was accompanied by the increased accumulation of several lymphoid populations and the local tumoricidal activity of CD8^+^ T cells. *In vitro* analysis revealed that MPO can associate with human T cells and negatively regulate their proliferation and activity. This study sheds light on a novel role of neutrophil-derived molecules as key players in tumor growth and, for the first time, identifies MPO as an enzyme within the TME with an immunosuppressive role in cancer.

## 2 Results

### 2.1 MPO expression is associated with shorter survival in patients with NSCLC and positively influences tumor growth *in vivo*

**Fig. 1:**
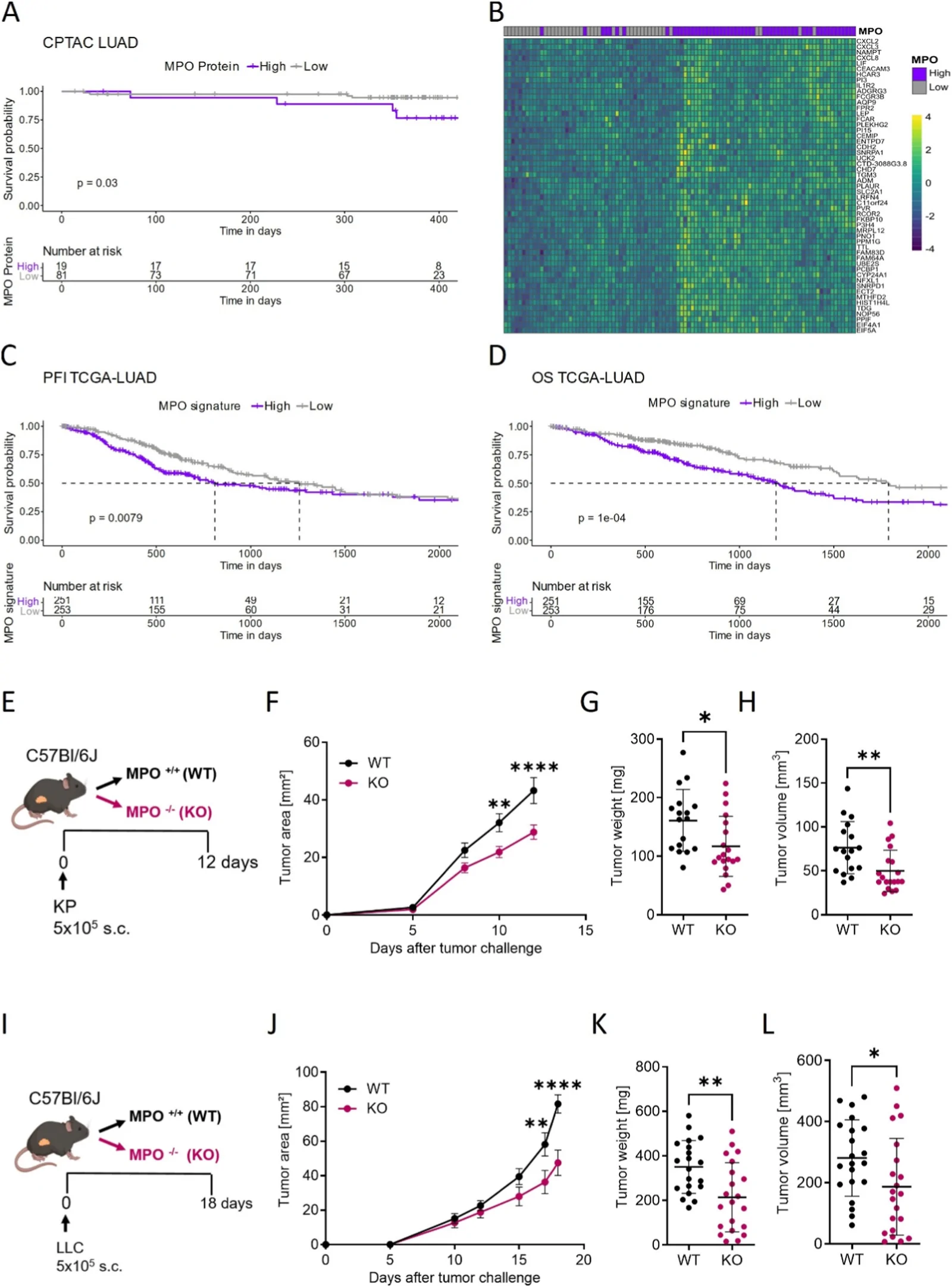
**MPO expression is clinically relevant in patients with NSCLC and impacts tumor growth in mice.** **(A)** Kaplan–Meier analysis of survival outcome differences in patients with NSCLC and different MPO expression levels. **(B)** Heatmap of differentially expressed genes in MPO-high and -low subgroups. The horizontal axis represents samples, and the vertical axis represents different genes. **(C)** Progression-free survival in TCGA patients divided into MPO-high and -low subgroups. **(D)** Overall survival in TCGA patients stratified by MPO expression. **(E and I)** Wildtype (WT) and MPO knockout (KO) mice were subcutaneously injected with 5 × 10^5^ kP or LLC cells, and tumors were allowed to grow for 12 or 18 days, respectively. **(F and J)** Tumor growth was monitored throughout the experiment. Data are presented as means ± SEM (n = 18-21). Statistical differences were determined using two-way ANOVA followed by Sidak's post hoc test. (**p < 0.01 and ****p < 0.0001). **(G, H, K, and L)** Mice were sacrificed, and tumor weight and volume were measured *ex vivo*. Data are presented as means ± SD (n = 18-21). Statistical differences were determined by unpaired Student's *t*-test (*p < 0.05 and **p < 0.01).

To determine the significance of MPO in NSCLC, the prognostic value of MPO protein expression in NSCLC was first investigated. Using the Clinical Proteomic Tumor Analysis Consortium (CPTAC) database, the influence of MPO protein expression in primary tumor tissues on survival was investigated using Kaplan–Meier analysis, revealing a benefit for patients with low MPO protein expression [Fig. 1(A)]. To further investigate the effect of MPO, patients were divided into MPO-high and -low groups based on the median MPO protein expression, and differentially expressed genes were determined using Welch's *t*-test with a cutoff of 0.05 for the adjusted *p*-values [Fig. 1(B)–Table S1]. In the TCGA-lung adenocarcinoma (LUAD) dataset, significantly upregulated genes were used to calculate an MPO signature score [Fig. 1(B)]. To validate the use of this signature, the scores of significantly upregulated and downregulated genes were correlated with each other, revealing a strong negative correlation, implying a biological reason driving this expression profile [Fig. S1(B) and (C)]. The effect of the signature on the progression-free interval (PFI) and overall survival (OS) was evaluated and revealed a higher survival in the low MPO signature group [Fig. 1(C) and (D)].

To evaluate how MPO deficiency affects tumor burden in mice, KP cells (isolated from a Kras^LSL−G12D^/p53 ^fl/fl^ mouse LUAD) or LLC cells (Lewis lung carcinoma) were injected subcutaneously into the flanks of C57BL/6 MPO^+/+^ WT and age-matched MPO-KO (MPO^−/−^) mice [Fig. 1(E) and (I)]. Tumor growth was monitored throughout the experiments. Growth curves revealed that KP and LLC tumors grew more slowly in MPO-KO animals than in WT animals [Fig. 1(F) and (J)]. *Ex vivo* analysis of the weight and volume of KP [Fig. 1(G) and (H)] and LLC [Fig. 1(K) and (L)] tumors revealed a 35% and 40% reduction in tumor size in MPO-KO mice, respectively. To confirm MPO-KO, MPO content was measured in homogenates from KP and LLC tumors through ELISA [Fig. S2(A) and (B)].

Our findings suggest that MPO plays a role in the development of NSCLC and has an impact on patient survival.

### 2.2 MPO deficiency favors an anti-tumorigenic immune cell profile in NSCLC mouse models

**Fig. 2:**
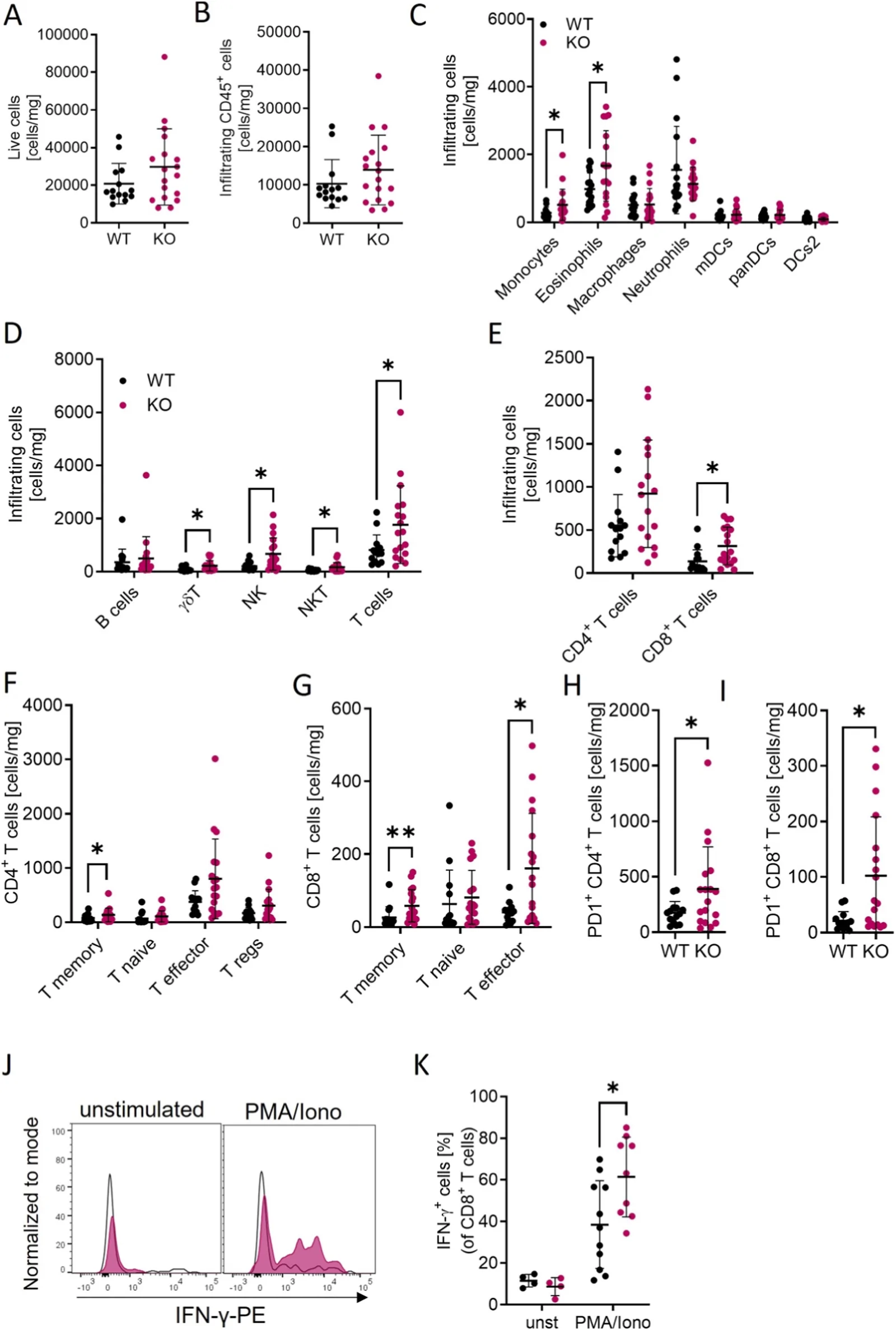
**MPO deletion favors lymphocyte infiltration in an engraftment lung cancer model.** **(A–I)** Flow cytometric analysis of single-cell suspensions of KP tumors from MPO wildtype (WT) and knockout (KO) mice. **(J)** Representative histograms of IFN-γ expression in tumor-infiltrating CD8^+^ T cells before (unstimulated) and after PMA/Iono stimulation. **(K)** Quantitative analysis of tumor-infiltrating CD8^+^ T cells revealed increased IFN-γ expression in MPO-KO mice after *ex vivo* PMA/Iono stimulation. DCs, dendritic cells; mDCs, myeloid dendritic cells; panDC, plasmacytoid DCs; iono, ionomycin; PMA, phorbol 12-myristate 13-acetate/ionomycin; NK, natural killer cells; NKT, natural killer T cells. Data are presented as means ± SD (n = 18-20). Statistical differences were determined using unpaired Student's *t*-test (*p < 0.05).

To determine whether the reduced tumor size observed in MPO-KO mice was accompanied by changes in the TME's immune cell profile, flow cytometry was used to determine the comprehensive profile of infiltrating immune cells and their subtypes in KP and LLC tumors [gating strategies shown in Fig. S3(A) and (B)]. There were no differences in the number of live cells and infiltrating leukocytes (CD45^+^ cells) in KP tumors from MPO-KO and WT mice [Fig. 2(A) and (B)]. MPO-KO mice had more monocytes and eosinophils than WT mice [Fig. 2(C)], but no changes in the other myeloid cell populations, including neutrophils, macrophages, dendritic cells (DCs), myeloid dendritic cells (mDCs) and plasmacytoid dendritic cells (panDCs) [Fig. 2(C)]. Additionally, we observed a significant shift in lymphoid cell populations in MPO-KO tumors compared to WT mice, with γδT cells, natural killer (NK) cells, natural killer T (NKT) cells, T cells (CD3^+^), and CD8^+^ T cells being increased in MPO-KO mice [Fig. 2(D) and (E)]. Interestingly, the relative abundance of memory CD4^+^ and CD8^+^ T cells, as well as effector CD8^+^ T cells, was higher in MPO-KO mice than in WT mice [Fig. 2(F) and (G)]. The expression of the inhibitory checkpoint receptor PD-1 on tumor-infiltrating CD4^+^ and CD8^+^ T cells was increased in tumors from MPO-KO mice, suggesting increased immune activity or possible T cell exhaustion of these cells [Fig. 2(H) and (I)]. MPO-KO mice had more live and CD45^+^ cells in their LLC tumors than WT mice [Fig. S4(A) and (B)]. Furthermore, B cells, NK cells, NKT cells, CD3^+^ T cells, CD4^+^ T cells, and CD8^+^ T cells were also increased [Fig. S4(C) and (D)]. The relative abundance of naïve and effector CD8^+^ T cells was higher in MPO-KO mice [Fig. S4(F)]. The expression of the inhibitory checkpoint receptor PD-1 on tumor-infiltrating CD8^+^ T cells was also increased in MPO-KO mice [Fig. S4(H)].

To assess the baseline immune composition of MPO-KO mice, WT and MPO-KO mice were compared, and no differences were observed in any of the immune populations in the spleens of nontumor-bearing [Fig. S5(A)–(E)] or tumor-bearing mice [Fig. S7(A)–(E)]. The thymus of tumor-free mice revealed a slight increase in the CD8^+^ single-positive thymocyte subpopulation (CD8^+^ SP) in MPO-KO mice compared to WT mice [Fig. S6(C)]. When compared to the WT, cells of the differentiation stage DN1 were reduced in the MPO-KO while cells of the differentiation stage DN4 were increased [Fig. S6(D)]. There were no differences in the other thymocyte populations [Fig. S6(A)–(D)].

To test whether T cells were more activated in MPO-KO mice, tumor-infiltrating CD8^+^ T cells from MPO-KO and WT mice were stimulated *ex vivo* with phorbol 12-myristate 13-acetate/ionomycin (PMA/Iono), and their activity was assessed using flow cytometry. MPO-KO tumors expressed higher IFN-γ levels on CD8^+^ T cells than WT mice [Fig. 2(J) and (K)], indicating local activation and enhanced tumoricidal activity of T cells in MPO-KO mice. Our findings, therefore, suggest that MPO depletion increases lymphocyte infiltration into the tumors.

### 2.3 Reduction of tumor size in MPO-KO mice is dependent on CD8^+^ T cells

**Fig. 3:**
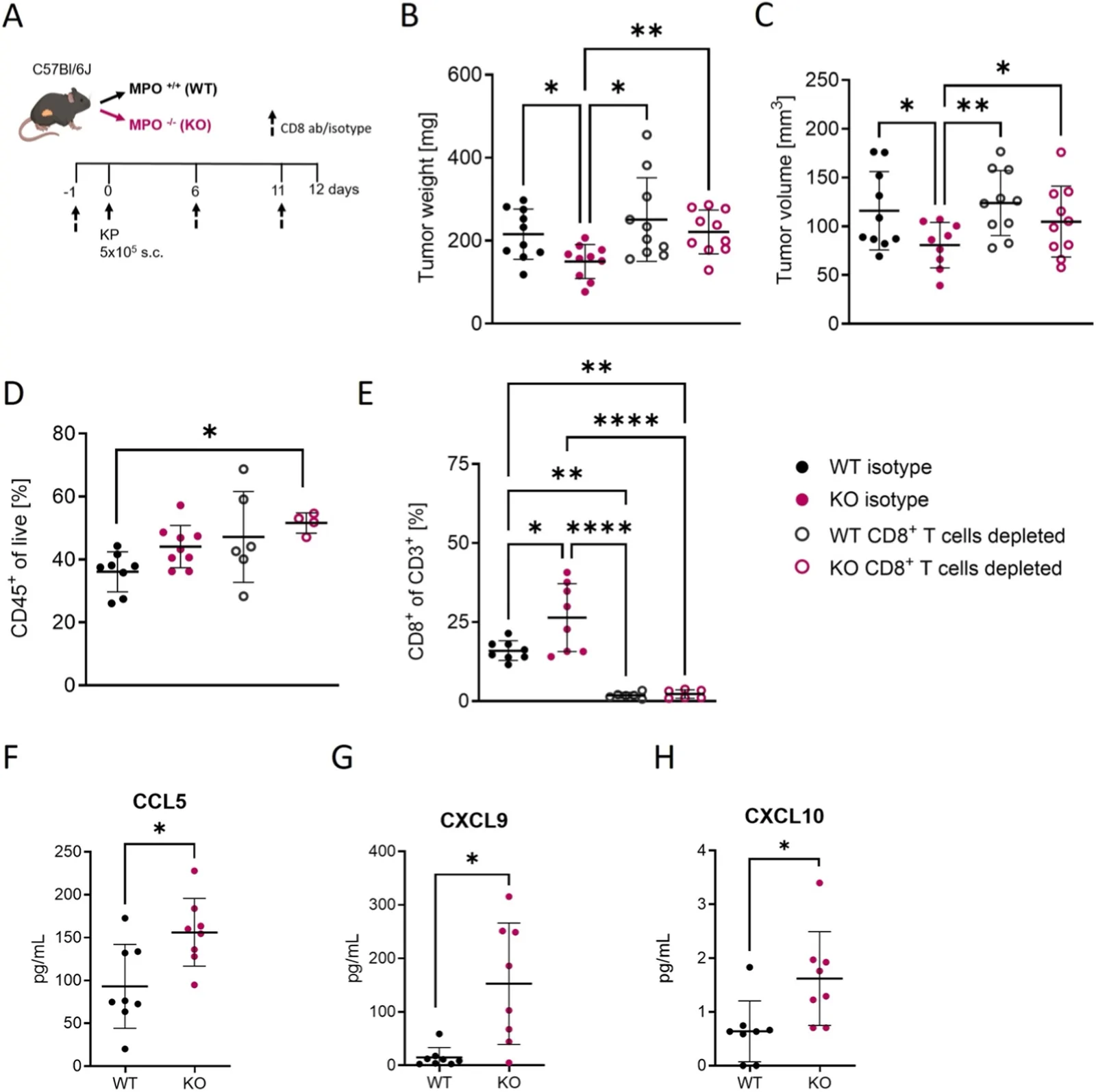
**CD8^+^ T cells are necessary to reduce tumor growth in MPO-KO mice.** **(A)** Schematic representation of the subcutaneous tumor model used in WT and MPO-KO mice. Anti-CD8 antibody (500 μg per mouse) was given intraperitoneally on days −1 (1 day prior to tumor cell injection) and 6, and 250 μg of anti-CD8 antibody was given intraperitoneally on day 11. **(B and C)** One day after the last anti-CD8 antibody injection, mice were sacrificed, and tumor weight and volume were measured *ex vivo* (n = 10). **(D and E)** Flow cytometric analysis of single-cell suspensions from tumors in WT and MPO-KO mice (n = 6-10). **(F-H)** LEGENDplex analysis of tumor homogenates from WT and MPO-KO mice (n = 8). Data are presented as means ± SD. Statistical differences were determined using unpaired Student's *t*-test (B, C, F-H) or one-way ANOVA followed by Tukey's multiple-comparisons test (D, E) (*p < 0.05, **p < 0.01 and ****p < 0.0001).

Given the differences in CD8^+^ T cell counts, KP tumor-bearing mice were injected intraperitoneally with anti-CD8 antibodies or control IgG isotypes to explore whether the reduction in tumor size in the MPO-KO mice is dependent on the presence of CD8^+^ T cells [Fig. 3(A)]. At the endpoint, tumors were harvested, weighed, measured, and processed for flow cytometry. The peripheral CD8^+^ T cell pool was depleted by 90% on average (Fig. S8(B)). In the isotype-treated mice, tumor weight and volume were reduced in the MPO-KO group vs the WT [Fig. 3(B) and (C)]. Interestingly, in the anti-CD8 antibody-treated mice, no differences in tumor weight and volume were observed between the WT and MPO-KO groups [Fig. 3(B) and (C)]. Flow cytometric analysis of tumor single-cell suspensions revealed no differences in CD45^+^ infiltration between WT and MPO-KO mice in both the isotype and anti-CD8 antibody groups [Fig. 3(D)]. As previously found, CD8^+^ T cells were significantly more abundant in isotype-treated MPO-KO mice than in WT mice [Fig. 3(E)]. In addition, CD3^+^ infiltration was reduced in the anti-CD8 antibody groups of WT and MPO-KO mice, while CD4^+^ T cells increased within the T cell compartment as expected [Fig. S9(A)–(E)]. LEGENDplex analysis of tumor homogenates revealed that MPO-KO tumors have higher expression of chemokines CCL5, CXCL9 and CXCL10 when compared to WT tumors [Fig. 3(F)–(H)]. Our findings suggest that CD8^+^ T cells are required for MPO-KO-dependent tumor growth reduction.

### 2.4 MPO decreases CD8^+^ T cell proliferation, function and associated with T cells *in vitro*

**Fig. 4:**
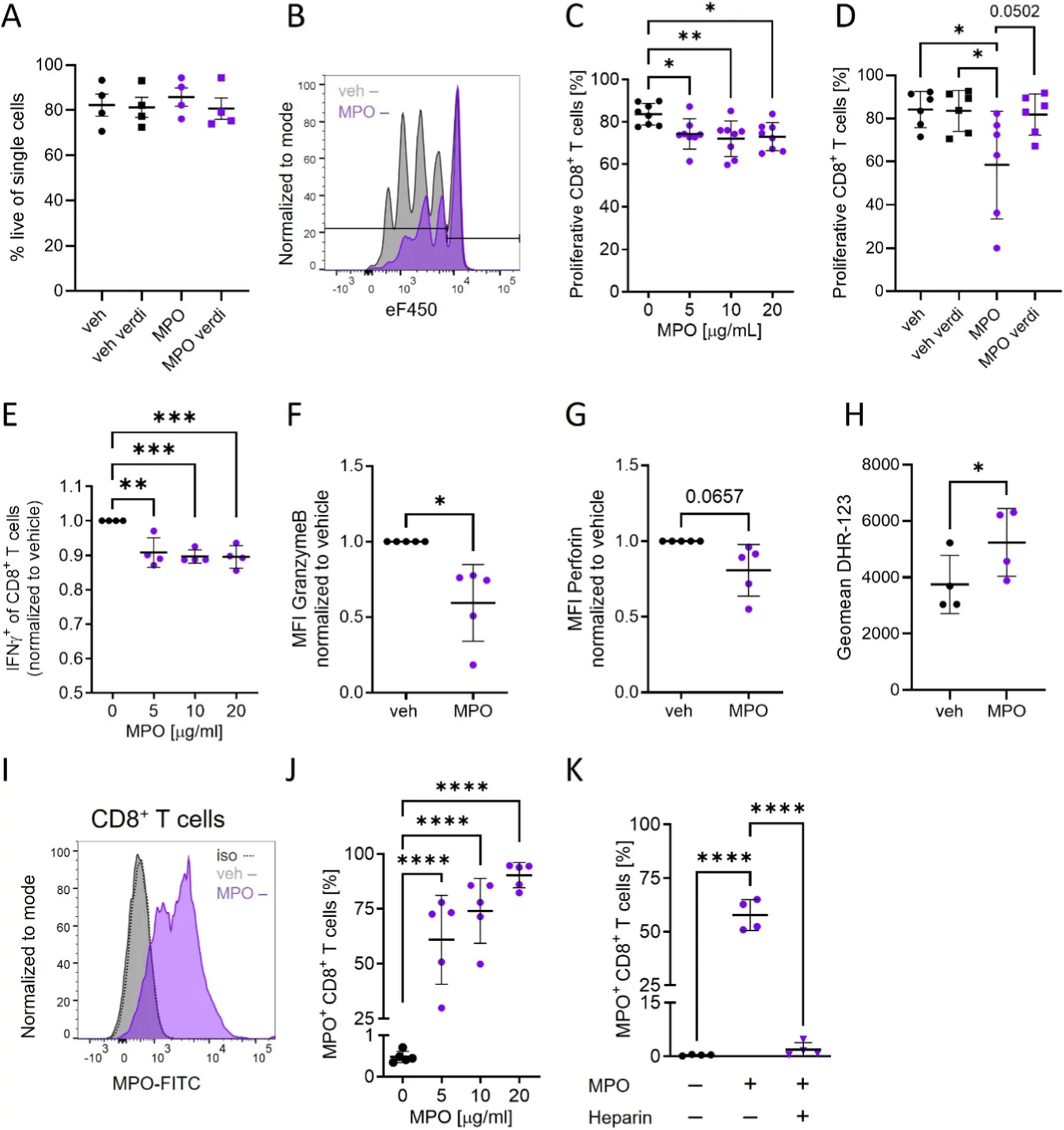
**MPO binds to CD8^+^ T cells and inhibits their proliferation and function *in vitro*** PBMCs were isolated from healthy donors, and the T cell fraction was enriched using negative selection. **(A–D)** 2.5 × 10^5^ T cells were labeled with the proliferation dye eFlourTM 450, treated with MPO **(C)**, or 5 μg/ml MPO and 10 μM verdiperstat (verdi) **(A and D),** and cultured for 72 h. Cells were checked for viability **(A)**, stained for CD8, and cell proliferation was evaluated as an increase in the percentage of dividing cells (n = 6-8). **(E)** 2.5 × 10^5^ T cells were incubated with MPO for 24 h. To stimulate IFN-γ production, cells were treated with ionomycin (1 μg/ml) and PMA (100 ng/ml). In addition, Golgi stop (1,5 μg/ml) was used to prevent IFN-γ secretion. After incubation, cells were stained for CD8, and IFNγ expression was evaluated using flow cytometry (n = 4). **(F and G)** 2.5 × 10^5^ T cells were incubated with 5 μg/ml MPO for 72 h, and intracellular staining of CD8, granzyme B and perforin was performed (n = 5). **(H)** 2.5 × 10^5^ T cells were activated overnight, incubated with 5 μg/ml MPO for 2 h and stained with DHR123 (n = 4). **(I and J)** T cells were treated with MPO for 2 h, and intracellular/nuclear flow cytometric staining was performed (n = 5). **(K)** Pretreated with heparin prior to MPO treatment (10 μg/ml) for 2 h followed by intracellular/nuclear flow cytometric staining was performed (n = 4). Data are presented as means ± SD. Statistical differences were determined using paired Student's *t*-test (E-G) or one-way ANOVA followed by Sidak's (B, D, I) or Tukey's (C, J) multiple-comparisons test (*p < 0.05, **p < 0.01, ***p < 0.001 and ****p < 0.0001).

Since tumors in MPO-KO mice had higher T cell infiltration and tumor reduction in MPO-KO mice was dependent on CD8^+^ T cells, the underlying mechanisms by which MPO could affect T cell behavior were investigated *in vitro*. T cells were isolated from healthy donors’ PBMCs, stained with the proliferation dye eFlour™ 450, and incubated with MPO for 72 h. MPO did not change overall cell viability [Fig. 4(A)]. CD8^+^ T cell proliferation was significantly reduced after 72 h incubation with MPO [Fig. 4(B) and (C)]. The MPO inhibitor verdiperstat could not significantly reverse this reduction in proliferation [Fig. 4(D)]. To investigate the effect of MPO on T cell activation, human CD8^+^ were treated with MPO (0, 5, 10, 20 μg/ml) for 24 h, stimulated with PMA/Iono, and assessed for activity as measured by their ability to produce IFN-γ using flow cytometry. MPO-treated CD8^+^ T cells showed decreased IFN-γ expression levels compared to vehicle-treated cells [Fig. 4(E)], indicating a reduced T cell activation. In addition, human T cells were treated with MPO for 72 h and cytotoxic markers granzyme B and perforin were assessed using flow cytometry. CD8^+^ T cells treated with MPO show reduced expression of both granzyme B and perforin compared to vehicle-treated cells [Fig. 4(F) and (G)]. To determine the effect of MPO on reactive oxygen species (ROS) levels, T cells were treated with MPO for 2 h and stained with DHR123 [Fig. 4(H)]. MPO-treated CD8^+^ T cells exhibited increased oxidized DHR123 levels compared with untreated controls. These findings imply that MPO has a direct impact on T cell function by reducing proliferation and activation.

Although MPO is not expressed by T cells, several studies have reported its ability to bind and internalize into endothelial and epithelial cells [[30], [31], [32]]. Accordingly, to investigate MPO binding to T cells, cells were incubated for 2 h in the presence of MPO (0, 5, 10, and 20 μg/ml), and MPO binding were assessed by flow cytometry. After 2 h of treatment, more than 50% of CD8^+^ T cells were MPO-positive [Fig. 4(I) and (J)]. It was previously shown that MPO binding is dependent on cell surface glycosaminoglycan interactions, and soluble glycosaminoglycans such as heparin can block MPO cellular uptake [30]. Heparin pretreatment for 45 min completely blocked MPO binding to T cells [Fig. 4(K)].

### 2.5 MPO is found in the lymphocytes of tumor samples from patients with NSCLC

**Fig. 5:**
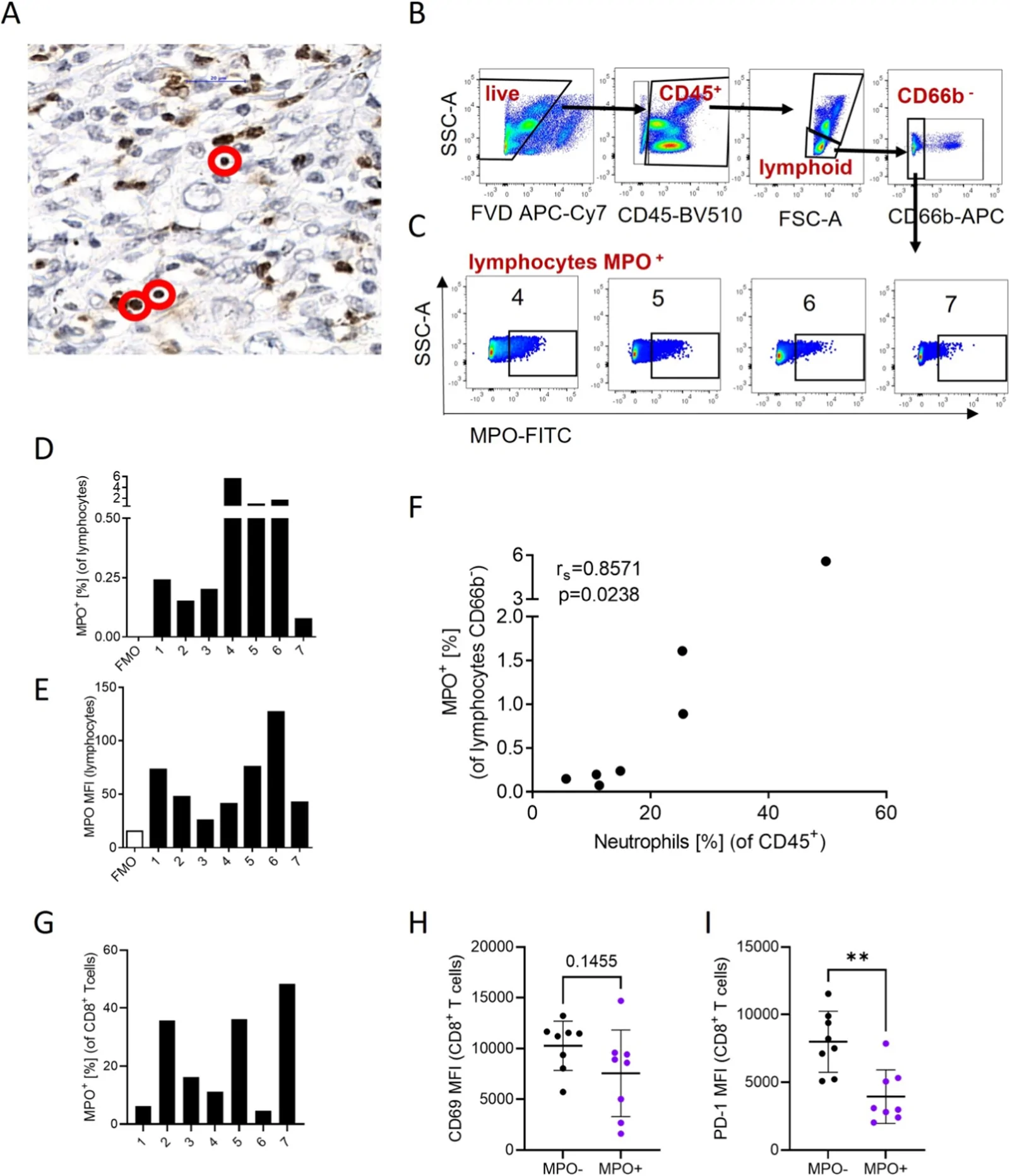
**MPO is found in lymphocytes from NSCLC tumor samples.** **(A)** MPO immunohistochemistry staining of tumor tissue, including lymphocytes (circle), shown at x80 digital magnification. **(B)** Representative flow cytometric dot plots demonstrating the gating strategy used to identify MPO^+^ signal in lymphocytes from patients with NSCLC. The initial gate is on live (FVD^−^) and CD45^+^ cells. The lymphoid population (T, B, and NK cells) was gated based on its forward and side scatter properties. The CD66b marker was used to exclude myeloid cells within this gate. **(C and D)** MPO^+^ lymphocytes in NSCLC tumor tissue and **(E)** MPO median fluorescence intensity of lymphocytes. **(F)** Correlations between MPO^+^ lymphocytes and neutrophil content in tumors from seven patients with NSCLC. The correlation was determined using Spearman's correlation coefficient rho (rs). **(G)** MPO^+^ CD8^+^ T cells in NSCLC tumor tissue. **(H and I)** Expression of CD69 and PD-1 in MPO^+^ CD8^+^ T cells in tumor tissue from NSCLC patients compared to MPO^−^ CD8^+^ T cells. Data are presented as means ± SD (n = 8). Statistical differences were determined using paired Student's *t*-test.

To investigate whether our findings about MPO binding to T cells was replicated in clinical samples, we stained human LUAD tissue sections and assessed NSCLC tissues for the presence of MPO in lymphocytes, including CD8^+^ T cells, by flow cytometry. Immunohistochemistry staining of LUAD tissues revealed the presence of MPO in lymphocytes, which is consistent with our *in vitro* findings [Fig. 5(A)]. Accordingly, flow cytometry revealed a lymphocyte MPO^+^ population in several NSCLC samples [Fig. 5(B)–(E)]. Interestingly, a positive correlation was found between the percentages of infiltrated neutrophils [defined as CD163^−^ CD66b^+^, Fig. S3(D)], the main source of MPO in NSCLC, and the percentages of MPO^+^ lymphocytes in NSCLC samples [Fig. 5(F)]. Detailed analysis of NSCLC samples revealed that tumor-infiltrating MPO^+^ CD8^+^ T cells [Fig. 5(G)] displayed a trend toward reduced CD69 expression [Fig. 5(H)], while PD-1 expression was significantly reduced compared to MPO^−^ CD8^+^ T cells [Fig. 5(I)]. These findings suggest that MPO-associated CD8^+^ T cells may exhibit altered activation within the TME.

## 3 Discussion

The importance of the TME as a niche that not only contains different cell types that modulate tumor growth but also delivers vital factors that support the escape from host immune surveillance and thus promote cancer development is well described [33]. In this context, neutrophils and neutrophil-derived molecules have been identified as important players in NSCLC development [15]. Molecules like neutrophil elastase have been shown to play a pro-tumorigenic role in NSCLC [20]. Accordingly, we aimed to determine whether another important neutrophil-derived granular enzyme, MPO, contributes to NSCLC carcinogenesis.

MPO is a heme peroxidase mainly expressed by neutrophils [22] but is also found in small amounts in monocytes and macrophages [27]. MPO is normally stored in neutrophil primary granules, but it can be rapidly released into the extracellular milieu in response to neutrophil activation, apoptosis, and necrosis. MPO's physiological roles have traditionally been described in the context of an innate immune response, in which this enzyme is responsible for pathogen killing once neutrophils arrive at the site of infection [34]. Recently, emerging roles of the enzyme have been identified, particularly in the context of cardiovascular disease and atherosclerosis, where MPO has been linked to both the initiation and progression of cardiac pathologies [35,36]. Since then, special interest has emerged in studying the role of MPO in other inflammatory diseases such as rheumatoid arthritis, cutaneous and pulmonary inflammation, and cancer [37].

Patients with NSCLC have been reported to exhibit elevated serum MPO levels compared with healthy individuals [38]. In the present study, patients with NSCLC and low MPO protein expression exhibited a significantly better survival prognosis than those with high MPO expression. This clinical distinction underscores the context-dependent role of MPO; while it serves as a positive prognostic differentiation marker in leukemia blasts [39], in solid tumors like NSCLC it acts as an extrinsic, neutrophil-derived mediator that drives an immunosuppressive TME.

Because MPO gene expression is restricted to neutrophil precursors in the bone marrow [40], MPO expression in solid tumor RNA datasets does not correlate with MPO protein expression. To address this limitation, the MPO signature was calculated to exploit MPO in publicly available gene expression data. The MPO-low signature was linked to better survival (PFI and OS). Consistent with our findings, higher MPO expression has been linked to increased oxidative stress and higher susceptibility to lung [41], ovary [42], and breast [43] cancer, among others. Furthermore, some MPO-derived oxidants can induce carcinogenesis by inducing DNA damage [44].

Using a syngeneic lung tumor model, we observed that tumors grown in MPO-deficient mice were smaller than those grown in WT mice. Our findings align with those of Rymaszewski et al., who demonstrated that MPO-KO mice exhibit reduced tumor burden compared to wildtype controls [45]. In their study, early administration of an MPO inhibitor in an inflammation-driven lung cancer model markedly impaired tumor growth, pointing toward a significant immunoregulatory role [45]. Consistently, we have previously reported that pharmacological MPO inhibition via 4-ABAH similarly attenuates tumor growth [46].

Interestingly, our study discovered that the immune cell landscape of MPO-KO tumors had shifted to a more anti-tumorigenic profile. Significant increases in lymphoid populations, including natural killer (NK), natural killer T (NKT) cells, γδ T cells, and CD3^+^CD8^+^ T cells were observed in MPO-KO mouse tumors compared to their WT counterparts. In line with our findings, increased immune infiltration of cytotoxic T cells such as CD3^+^CD8^+^ T cells and NK cells has been linked to a better prognosis for lung cancer [47,48]. A more in-depth investigation of CD8^+^ T cells revealed that they were also more active with higher IFN-γ levels. Previous reports found that patients with NSCLC had a lower number of cytotoxic T cells as well as IFN-γ expression [49,50]. Hence, MPO deficiency may contribute to tumor shrinkage by improving the local immune microenvironment and boosting the activity of infiltrated lymphocytes. Further analysis of CD8^+^ T cell subpopulations revealed an increase in effector and memory CD8^+^ T cells, which is consistent with previous studies that found a reduction in T effector cell infiltration in patients with NSCLC [50]. Another interesting finding was that PD-1 expression was higher in CD4^+^ and CD8^+^ lymphocytes from MPO-KO mice, suggesting enhanced T cell activity against tumor antigens or possible T cell exhaustion, and potentially serving as a predictor of response to anti-PD-1 therapy [51,52]. Interestingly, another study found that using an MPO inhibitor in combination with PD-1 checkpoint blockade reduced tumor progression [53] pointing to MPO as a potential therapeutic target to improve current cancer immunotherapies.

Cytotoxic CD8^+^ T cells play an important role in the anticancer immune response [4]. To determine whether the increased number of CD8^+^ T cells was linked to a reduced tumor burden in MPO-KO mice, anti-CD8 antibodies were used to deplete CD8^+^ T cells. Remarkably, no differences in tumor weight or volume were observed between the WT and MPO-KO groups when CD8^+^ T cells were abolished. Thus, the impact on the CD8^+^ T cell population may represent an important mechanism against tumorigenesis caused by MPO deficiency in the TME. T cell recruitment to the TME is largely regulated by chemokine signaling [54]. In our study, tumors from MPO-deficient mice exhibited increased levels of CCL5, CXCL9, and CXCL10 compared to wildtype controls. These chemokines have all previously been identified as chemoattractants for CD8^+^ T cells and have been shown to promote the recruitment of CD8^+^ T cells in NSCLC [[55], [56], [57]]. This suggests that MPO deficiency may alter the chemokine landscape of the TME, potentially enhancing T cell infiltration. Previous murine studies in melanoma and pancreatic cancer models have demonstrated that deletion or inhibition of MPO enhances response to immune checkpoint blockade, leading to reduced tumor growth [28,29]. These findings are consistent with our results However, our data extend these observations by linking reduced tumor growth with altered CD8^+^ T cell function, providing functional insight into the immunomodulatory role of MPO within the TME.

Tumor cells produce cytokines and chemokines that attract leukocytes, including T lymphocytes, to the tumor site. Human CD8^+^ T cells treated with MPO showed reduced proliferation and IFN-γ production *in vitro*, while cell viability remained unaffected. In addition, expression of the cytotoxic marker granzyme B was reduced in MPO-treated CD8^+^ T cells, while MPO exposure increased intracellular ROS levels. These findings indicate that MPO directly alters T cell behavior. Other studies have shown that after antigen injection, neutrophils rapidly infiltrate draining lymph nodes, where MPO is released [58]. The deposited MPO suppresses dendritic cell (DC) function and migration, resulting in a decrease in CD4^+^ T cell responses, including T cell activation, proliferation, and differentiation into Th1 and Th17 effectors [58]. Accordingly, MPO-driven lipid peroxidation has recently been described as a mechanism by which MPO impairs antigen cross-presentation by DCs in tumors [53].

MPO is a highly cationic molecule that rapidly binds to negatively charged structures such as bacterial surfaces, extracellular matrix components, and cellular membranes, including those of endothelial cells and neutrophils themselves [30,59]. MPO remodels the endothelial transcriptional landscape by translocating into endothelial nuclei, binding and decondensing chromatin [32]. Moreover, extracellular MPO uptake has been reported in neutrophils and macrophages via mechanisms involving CD11b/CD18 integrins and the mannose receptor, respectively [60,61]. MPO uptake has previously been demonstrated in endothelial cells, and a functional impact of MPO after binding and internalization has also been reported [61]. Accordingly, we found that MPO could bind to CD8^+^ T cells, and that heparin pretreatment completely blocked the presence of MPO in T cells. This is consistent with another study that found the similar effect in endothelial cells [30]. Finally, we identified MPO^+^ lymphocytes in tumors from NSCLC patients, and the abundance of MPO^+^ lymphocytes positively correlated with neutrophil content. Moreover, MPO^+^ CD8^+^ T cells in NSCLC tumor tissue exhibited reduced activation, as indicated by lower CD69 and PD-1 expression, compared with MPO^−^ CD8^+^ T cells. These findings suggest that MPO uptake may alter T cell function within the TME. Further studies are needed to elucidate the mechanism by which MPO impairs T cell function.

In conclusion, our findings suggest that MPO in the NSCLC TME may act as an immunosuppressive molecule, impairing T cell behavior and thus promoting tumor growth. This supports the potential clinical targeting of MPO in patients with NSCLC, particularly those with high neutrophil content in the TME. By inhibiting MPO activity, the TME may become more permissive to T cell infiltration and activation, potentially overcoming the resistance often seen in immunologically cold NSCLC tumors.

## 4 Materials and methods

### 4.1 Bioinformatics

CPTAC cohort: CPTAC LUAD protein, transcript, clinical, and survival data were accessed using the cptac python package (https://pypi.org/project/cptac/). Patients were divided into groups based on the median MPO protein expression, and Welch's *t*-test was used to identify differentially expressed genes. A critical *p*-value of 0.05 was chosen, and Bonferroni correction was used as an adjustment method. Significantly upregulated genes were plotted on a heatmap using the R package pheatmap, and this list of genes was further termed the “MPO signature.” Significantly downregulated genes were termed the “control signature” and were used as a control in other datasets to assess the plausibility of the MPO signature. MPO protein and transcript expressions were correlated using Pearson's correlation and plotted using the R package ggpubr. The survival curve was created using the R package survminer. The cutpoint was determined using the surv_cutpoint function of this package.

The Cancer Genome Atlas (TCGA)-LUAD cohort: Transcriptomic (TPM), clinical, and survival data were accessed via the pan-cancer atlas hub on the UCSC Xena browser. The MPO signature was calculated using the z-score method in the R package hacksig. The Kaplan–Meier analysis was carried out using the R package survminer, and the median expression of the MPO signature was used as a cutpoint.

ICP3 cohort: The MPO signature was calculated using the z-score method in the R package hacksig and correlated with T cell content (% CD45^+^) derived from flow cytometry [15].

MPO signature plausibility check: In datasets where the MPO signature was used, MPO signature and control scores were scaled using R's scale function and correlated using Pearson's correlation. In the ICP3 cohort, the neutrophil content determined by flow cytometry was correlated with the scaled MPO signature score. In the TCGA-LUAD cohort, the neutrophil content obtained from the Kassandra website (https://science.bostongene.com/kassandra/downloads, 10.1016/j.ccell.2022.07.006) was correlated with the scaled MPO signature score.

Software: Python 3.10.6, R 4.2.1, Platform: x86_64-conda-linux-gnu (64-bit), operating system: Debian GNU/Linux 11 (bullseye).

### 4.2 Animal studies and cell culture

All animal experiments were performed at the animal facilities of the Medical University of Graz. The experimental protocols were approved by the Austrian Federal Ministry of Science and Research (BMWF-66.010/0041-V/3b/2018). MPO^−/−^ mice (B6.129X1-Mpo^tm1Lus^/J) were obtained from the Jackson Laboratory (Bar Harbor, ME, USA) and backcrossed to the C57BL/6 J strain. Genotyping was performed from ear tags using standard PCR protocols. The murine KP cell line (generously provided by Dr. A. McGarry Houghton, FredHutch, Seattle) was isolated from a LUAD of a Kras^LSL−G12D^/p53 ^fl/fl^ mouse at the Fred Hutchinson Cancer Center (Seattle, WA, USA) after intratracheal administration of adenoviral Cre recombinase, as previously described [62]. The murine LLC cell line (ATCC® CRL-1642™) as well as the human LUAD cell line A549 (ATCC® CCL-185™) were supplied by ATCC. KP and LLC cells were cultured in Dulbecco's Modified Eagle Medium supplemented with 10% fetal bovine serum (FBS, Life Technologies; catalog nos. 21875–091 and 10270106, respectively) and 1% penicillin/streptomycin (P/S, PAA Laboratories; catalog no. P06-07100) and incubated at 37 °C and 5% CO_2_ in a humidified atmosphere.

### 4.3 Murine tumor models

For the heterotopic lung tumor engraftment model, KP or LLC cells were subcutaneously injected under inhaled isoflurane anesthesia. A total of 5 × 10^5^ kP or LLC cells suspended in 450 μl Dulbecco's phosphate-buffered saline (PBS, Gibco) were injected subcutaneously into the flanks of MPO-KO and WT mice aged 6–14 weeks. To deplete CD8 T cells *in vivo*, tumor-bearing MPO-KO mice and WT littermates were injected intraperitoneally with 500 μg of rat monoclonal antibody to mouse CD8α (clone YTS 169.4) (BioXCell; catalog no. BE0117) on Days −1 (1 day prior to tumor cell injection) and 6, and with 250 μg on Day 11, or with rat IgG2b isotype control antibody (Clone LTF-2, BioXCell; catalog no. BE0090). When tumors became palpable (after about 5 days), mice were shaved, and tumor growth was monitored and measured (length and width) every other day using a digital caliper throughout the experiment. Mice were sacrificed on Day 12 (KP cells) or Day 18 (LLC cells), and tumors were harvested, weighted, and measured with a digital caliper *ex vivo.* Tumor volume was calculated using the formula: v=length×width×height×π/6) [62]. Tumors were kept in Roswell Park Memorial Institute (RPMI) on ice until further use or were immediately fixed in 10% RotiR-Histofix or snap frozen in liquid nitrogen.

### 4.4 Human NSCLC tissue samples

Patients with NSCLC Stages IA-IIIB were recruited from the Departments of Internal Medicine, Oncology, and Surgery, Division of Thoracic Surgery, Medical University of Graz (Graz, Austria). Informed consent was obtained from all participants. The study adhered to the Helsinki Declaration and was approved by the Ethics Committee of the Medical University of Graz (MUG-EK-number: 30-105 ex17/18).

### 4.5 Single-cell suspensions

Single-cell suspensions from mouse and human tumors were prepared as previously described [62]. Tissue was minced with surgical scissors and digested with collagenase IV (CLS-1; 4.5 U/ml; Worthington) and DNase I (160 mU/mL, Worthington; LS002006) in RPMI for 20 min at 37 °C while rotating at 1000 rpm. Tissue digests were passed through a 100 μm strainer, enzymes were inhibited by adding staining buffer (SB, PBS+2% FBS), and centrifuged for 5 min at 500 ×g (4 °C). The pellet was resuspended in red blood cell lysis buffer (BioLegend, catalog no. 420301, all human samples) and incubated for 3 min on ice with periodic shaking. The lysis process was neutralized by adding 4 vol of PBS. The cells were then passed through a 40 μm strainer into a new 50 ml tube and washed in SB. Subsequently, cells were washed twice in PBS, counted using the EVE automated cell counter (NanoEnTek), and stained with surface and intracellular antigens.

### 4.6 Flow cytometry

Mouse: To exclude dead cells, single-cell suspensions were incubated for 20 min at 4 °C in the dark in Fixable Viability Dye (FVD) eFluorTM 780 (eBioscience; catalog no. 65-0865-14). After adding 1 μg mouse TruStain FcX™ (Biolegend; catalog no. 101320) for 10 min, immunostaining was performed for 30 min at 4 °C (protected from light) with the following antibodies (Table S1): CD45-AF700 (catalog no. 103128), CD45-BV785 (catalog no. 103149), Ly6C-APC (catalog no. 128015), Ly6G-PE/Dazzle594 (catalog no. 127648), CD11c-BV605 (catalog no. 117334), CD8-PerCPCy5.5 (catalog no. 100734), PD1-APC (catalog no. 135210), CD25-BV785 (catalog no. 102051) CD62L-BV605 (catalog no. 104438), NKp46-BV510 (catalog no. 137623), CD19-FITC (catalog no. 115506), PDL1-PECy7 (catalog no. 124313), CD206-FITC (catalog no. 141703), MHCII-PerCP-Cy5.5 (catalog no. 107625), CCR3-BV421 (catalog no. 144517), CD103-BV510 (catalog no. 121423) (all antibodies from Biolegend), and CD11b-BUV737 (catalog no. 612801), F4/80-BUV395 (catalog no. 565614), Siglec-F-PE (catalog no. 562068), CD3-BUV395 (catalog no. 563565), CD4-BUV496 (catalog no. 564667), CD44-BUV737 (catalog no. 612799), gdTCR-PECF594 (catalog no. 563532) (all antibodies from BD Biosciences), and FoxP3-PE (eBio, catalog no. 12-5773-82). For nuclear antigen staining, cells were permeabilized with Transcription Factor Buffer Set (BD Biosciences, catalog no. 562574) prior to staining procedures. To detect changes in interferon-gamma (IFN-γ), single-cell suspensions of tumors or spleens (2 × 10^6^ cells per well) were seeded into 96-well U-bottomed plates with RPMI containing 10% FBS, 1% P/S, and GolgiStop (1.5 μl/ml, BD Biosciences), and incubated for 4 h at 37 °C. During that time, they were either stimulated with phorbol 12-myristate 13-acetate/ionomycin (PMA, 100 ng/ml, Sigma-Aldrich) and ionomycin (1 μg/ml, Sigma-Aldrich) or left unstimulated. Subsequently, surface staining was performed with CD45-FITC (catalog no. 103108), CD3-BV421 (catalog no. 100336), CD4-PE-Cy7 (catalog no. 100528), and CD8-PerCP5.5 (catalog no. 100734), followed by intracellular labeling (BD Cytofix/CytopermTM Kit) with IFN-γ-PE (catalog no. 505808). The samples were analyzed on a BD LSRFortessa™ or a BD Canto™ flow cytometer with FACSDiva software (BD Biosciences, Franklin Lakes, NJ, USA) and over 2 × 10^5^ events per sample were recorded. The data were compensated and analyzed using FlowJo software (TreeStar, Ashland, OR, USA) and gates were defined using fluorescence-minus-one samples [Fig. S3(A)–(C)].

Human: The staining was performed as described above. In brief, FVD eFluor™ 780 (FVD, eBioscience) (20 min, 4 °C) was used to exclude dead cells. Cells were preincubated in SB (staining buffer: PBS containing 2% FBS) with human TruStain FcX blocking solution (FcBlock, Biolegend) at 4 °C for 10 min. Subsequently, cells were stained in SB for 20 min at 4 °C with CD45-BV510 (catalog no. 304035), CD163-BV605 (catalog no. 333616), CD66b-APC (catalog no. 305118), CD3-AF700 (catalog no. 300424), CD4-BUV395 (catalog no. 563550), CD8-BV605 (catalog no. 301040), CD69-BV421 (catalog no. 310930), PD1-BUV737 (catalog no. 612792) and MPO-FITC (catalog no. 333138) antibodies. Cells were centrifuged (500 ×g, 5 min, 4 °C), resuspended in 200 μL SB, and washed again with SB. Cells were fixed in IC fixation buffer (eBioscience, ThermoFisher) for 10 min at 4 °C, centrifuged, and resuspended in 100 μL SB. For intracellular staining, cells were permeabilized, fixated (BD Cytofix/CytopermTM Kit) and stained with IFN-γ-BUV395 (catalog no. 563563), Granzyme-B-FITC (catalog no. 515403), Perforin-PE (catalog no. 353304). For ROS production, cells were stained with dihydrorhodamine 123 (DHR123) (20 min, 37°C), washed in SB and measured without fixation. The samples were measured on a BD LSR II Fortessa (BD Biosciences).

### 4.7 Mouse tumor LEGENDplex

Small pieces of tumors from MPO-KO and WT mice were weighed and immediately frozen. Subsequently, homogenates were generated in PBS. Pierce™ BCA Protein Assay (Thermo Scientific) was used according to the manufacturer's instructions to determine protein concentration. A total of 40 μg of protein were applied in LEGENDplex™ Mouse Pro-inflammatory Chemokine Panel (Biolegend, catalog no. 740451). The assay was performed in a V-bottom plate and analyzed with the LEGENDplex software offered by the company following the manufacturer's instructions.

### 4.8 Mouse MPO ELISA

Frozen KP and LLC tumor tissue was homogenized using a Precellys 24 (Bertin Technologies) tissue homogenizer. Protein concentration was adjusted using Pierce BCA Protein assay (Thermo Scientific). MPO content in tumor homogenates was measured using Mouse Myeloperoxidase DuoSet ELISA (R&D systems #DY3667) according to the kit protocol.

### 4.9 Preparation of human peripheral blood leukocytes

Blood was drawn from healthy volunteers in accordance with a protocol approved by the Ethics Committee of the Medical University of Graz (EK-numbers: 17-291 ex 05/06), as previously described [63]. Blood (35 ml) was collected in a 50 ml sodium citrate-containing tube. Platelet-rich plasma was separated by centrifugation at 300 ×g for 20 min, and erythrocytes by dextran sedimentation. Dextran T-500 (Sigma-Aldrich; 6 ml, 6% in saline) was added to the cells and filled up to 50 ml with 0.9% saline. Dextran-crosslinked erythrocytes were allowed to sediment for 30 min, and the upper phase containing non-sedimented leukocytes was layered on top of 15 ml Histopaque (Sigma-Aldrich). High-density polymorphonuclear leukocytes, including neutrophils and eosinophils, were separated from less dense peripheral blood mononuclear cells (PBMCs), which included basophils, monocytes, lymphocytes, and dendritic cells, by centrifugation at 350 ×g for 20 min. The PBMCs were isolated from the interphase and washed in a Ca^2+^ and Mg^2+^-free assay buffer.

### 4.10 T cell isolation

T cells were enriched using the Easy Sep Human T Cell Isolation Kit (Stemcell Technologies) according to the manufacturer's instructions. Briefly, 50 × 10^6^ PBMCs/mL were resuspended in SB (PBS containing 2% FBS) and 1 mM EDTA, and then an isolation cocktail solution was added to the PBMCs and incubated for 5 min at room temperature. After incubation, RapidSphere beads were added, and the tube was placed in a magnet for 3 min at room temperature. The enriched sample was collected and washed with PBS. Live cells were counted using a Neubauer chamber and trypan blue. T cells were cultured in prewarmed RPMI media containing 1% P/S and were treated with different MPO (Elastin Products, catalog no. MY862) concentrations at various time points. For proliferation experiments, T cells were simultaneously treated with verdiperstat (MedChemExpress, catalog no. HY-17646).

### 4.11 T cell proliferation

T cells were labeled with eFluor™ 450 (eF450, 10 μM in PBS; 10 mio cells/mL; Thermo Fisher) for 10 min. Cells were washed twice with SB and resuspended in T cell proliferation medium (1% P/S, 50-μM β-mercaptoethanol, 2-mM l-Glutamine, 25-mM HEPES, 5-ng/ml interleukin-2, and 1-μg/ml αCD28) in X-VIVO 15 (Lonza). T cells (2.5 × 10^5^) were seeded in 96-well plates previously coated with αCD3 (5 μg/ml in PBS, 200 μl/well, overnight, 4 °C). Cells were incubated for 72 h at 37 °C. After incubation, cells were transferred to 5 ml FACS tubes, washed once with PBS, resuspended in 50 μL FcX-SB (0.5 μL/test), and incubated for 10 min (4 °C). Next, 50 μL of Ab-SB-solution (1 μL/test CD8-FITC) were added to the cells and incubated for 30 min. After washing twice with SB, cells were fixed with IC fixation buffer (Thermo Fisher Scientific) for 10 min at 4 °C, centrifuged, and resuspended in 100 μL SB. Samples were measured on a FACS Canto II (BD Biosciences).

### 4.12 Statistical analysis

Flow cytometric data were reported in cells/mg. Flow cytometric data compensation was performed using single stains. The “fluorescence-minus-one” strategy was used to calculate background fluorescence cutoffs [64]. Data were analyzed using the FlowJo software (TreeStar).

All statistical analyses were performed using GraphPad Prism 6.1 (GraphPad Software). All variables were tested for Gaussian distribution using the Shapiro–Wilk normality test. Significant differences between two experimental groups with normal distribution were determined using either paired or unpaired student's *t*-tests with Welch's correction; otherwise, the Mann–Whitney test was applied. Statistical comparisons among three or more groups were conducted using one-way analysis of variance (ANOVA), followed by Tukey's post hoc test for multiple pairwise comparisons or Sidak's post hoc test when comparing each group with its respective control. Tumor growth curves were analyzed using two-way ANOVA with Sidak's post hoc test. Neutrophil content and MPO^+^ lymphocytes were correlated using Pearson's correlation coefficient (r) and Spearman's correlation coefficient rho (r_s_). The results were expressed as the mean and standard error of the mean, or standard deviation. A *p*-value of <0.05 was considered statistically significant, and it was denoted with one, two, three, or four asterisks when it was <0.05, 0.01, 0.001, or 0.0001, respectively. Detailed description of the statistical tests used are provided in the respective figure legends.

## CRediT authorship contribution statement

**Paulina Valadez-Cosmes:** Writing – original draft, Visualization, Project administration, Methodology, Formal analysis, Data curation, Conceptualization. **Kathrin Maitz:** Writing – review & editing, Visualization, Validation, Project administration, Methodology, Formal analysis, Data curation, Conceptualization. **Anna Lagler:** Writing – review & editing, Visualization, Validation, Project administration, Methodology, Formal analysis, Data curation, Conceptualization. **Oliver Kindler:** Writing – review & editing, Visualization, Software, Resources, Methodology, Formal analysis. **Nejra Cosic Mujkanovic:** Writing – review & editing, Methodology, Formal analysis. **Sofia Raftopoulou:** Writing – review & editing, Methodology, Formal analysis. **Melanie Kienzl:** Writing – review & editing, Methodology, Formal analysis. **Zala Nikita Juvan:** Writing – review & editing, Methodology, Formal analysis. **Ana Santiso:** Writing – review & editing, Methodology, Formal analysis. **Luka Brcic:** Writing – review & editing, Methodology, Data curation. **Gregor Gorkiewicz:** Writing – review & editing, Methodology, Data curation. **Jörg Lindenmann:** Writing – review & editing, Resources. **Wolfgang Sattler:** Writing – review & editing, Resources. **Akos Heinemann:** Writing – review & editing, Resources. **Rudolf Schicho:** Writing – review & editing, Resources, Methodology. **Gunther Marsche:** Writing – review & editing, Resources, Methodology. **A. McGarry Houghton:** Writing – review & editing, Resources, Methodology. **Julia Kargl:** Writing – review & editing, Writing – original draft, Resources, Project administration, Methodology, Investigation, Funding acquisition, Data curation, Conceptualization.

## Ethics declaration

Written informed consent to take part in the study and to publish the article has been obtained from all participants or their legal representatives. The privacy rights of participants have been observed.

This study included organ or tissue donors. This study includes human biological material and consent was obtained by donors, or their next of kin or legal representatives, for use in this study and for publication of the article. The samples used in this research were not sourced from executed prisoners or prisoners of conscience.

This study was performed in compliance with relevant laws, regulatory frameworks and guidelines where the research took place. This study was approved by the Medical University of Graz. (Approval No. 30-105 ex17/18)

This study was conducted in accordance with the ARRIVE (Animal Research: Reporting of In Vivo Experiments) guidelines. This study was approved by the BMBWF. (Approval No. BMWF-66.010/0041-V/3b/2018)

## Declaration of generative AI and AI-assisted technologies in the writing process

During the preparation of this work, the authors used Gemini and ChatGPT to improve language clarity, readability and flow, as well as to assist with text editing. After using these tools, the authors reviewed and edited the content as needed and take full responsibility for the content of the published article.

## Funding

The lab of JK is supported by the OENB Anniversary Fund (17584), the FFG-Bridge 1 grant (871284), and the FWF-P35294 grant. Doctoral candidates are funded by the following doctoral programs FWF DK-MOLIN (W1241): PV-C, SR, AS; FWF doc-fund RespImmun, DOC-129: NCMand DP-iDP, DOC-31: ZNJ; OEAW Doc Fellowship - 26477: OK, and BioTechMed: MK. The lab work of RS is funded by the FWF grants P33325 and KLI887.

## Declaration of competing interest

The authors declare that they have no known competing financial interests or personal relationships that could have appeared to influence the work reported in this paper.

## Data Availability

Data will be made available on request.
