# Supplementary material for: Myeloperoxidase promotes a tumorigenic microenvironment in non-small cell lung cancer
Source: Redox Biol. 2026 Sep 15;97:104399. doi: 10.1016/j.redox.2026.104399 (PMC13602051; doi:10.1016/j.redox.2026.104399)
Supplement: Supplementary file 1 [file mmc1.docx]

**Supplementary Materials**

**Myeloperoxidase promotes a tumorigenic microenvironment in non-small cell lung cancer**

Paulina Valadez-Cosmes, Kathrin Maitz, Anna Lagler, Oliver Kindler, Nejra Cosic Mujkanovic, Sofia Raftopoulou, Melanie Kienzl, Zala Nikita Juvan, Ana Santiso, Luka Brcic, Gregor Gorkiewicz, Jörg Lindenmann, Wolfgang Sattler, Akos Heinemann^,^ Rudolf Schicho, Gunther Marsche, A. McGarry Houghton, and Julia Kargl

**Table of Contents**

Tables S1 and S2

Figures S1-S9

**Table S1. Genes derived from the CPTAC-LUAD dataset.**

| **MPO signature** | | **Control signature** | | | |
| --- | --- | --- | --- | --- | --- |
| ADGRG3 | HIST1H4L | ACOXL | CLK4 | LRRC31 | TLR5 |
| ADM | IL1R2 | ADGRF5 | CNKSR2 | MAP3K15 | TMEM243 |
| AQP9 | LEP | ADHFE1 | CREBRF | MAPK10 | TNN |
| C11orf24 | LIF | ANK3 | CRYM | MR1 | WWC1 |
| CDH2 | LRFN4 | ANKDD1B | CYP4V2 | MS4A2 | ZDHHC15 |
| CEACAM3 | MRPL12 | ANKRD44 | CYSLTR1 | MYOZ1 | ZMAT1 |
| CEMIP | MTHFD2 | AR | ETFBKMT | N4BP2L1 | ZNF441 |
| CHD7 | NAMPT | ARHGAP31 | FAM161B | NFIX | ZNF540 |
| CTD-3088G3.8 | NFXL1 | ARHGDIB | FAM184A | NIPAL3 | ZNF554 |
| CXCL2 | NOP56 | ATP13A4 | FMO4 | NPC2 | ZNF563 |
| CXCL3 | P3H4 | ATP13A5 | FMO5 | NRN1L | ZNF763 |
| CXCL8 | PCBP1 | B3GAT1 | GAB1 | PIGR | ZNF846 |
| CYP24A1 | PI15 | C16orf89 | GANC | POU2F3 | ZRSR2 |
| ECT2 | PI3 | C17orf50 | GPR160 | PPP2R3A |  |
| EIF4A1 | PLAUR | C1orf116 | GSAP | PTPN13 |  |
| EIF5A | PLEKHG2 | CA1 | HAS3 | RBL2 |  |
| ENTPD7 | PNO1 | CA13 | HLA-DPB1 | REPS2 |  |
| FAM64A | PPIF | CACNA1F | HLF | RNASE1 |  |
| FAM83D | PPM1G | CAMK2D | HNMT | RP11-490B18.9 | |
| FCAR | PVR | CAPN3 | HPGDS | SCTR |  |
| FCGR3B | RCOR2 | CARF | HSD17B6 | SGF29 |  |
| FKBP10 | SLC2A1 | CD1B | IL11RA | SLC15A2 |  |
| FPR2 | SNRPA1 | CD1E | IL6R | SLC9A5 |  |
| HCAR3 | SNRPD1 | CD302 | KAT2B | SLFN14 |  |
|  | TDG | CD40LG | KCNK5 | SMCO3 |  |
|  | TGM3 | CD74 | KIF13A | SOGA3 |  |
|  | TTL | CEBPA | KLHDC1 | TCEANC |  |
|  | UBE2S | CFAP221 | LANCL3 | TEF |  |
|  | UCK2 | CIRBP | LRP2BP | TLR3 |  |
| MPO Signature: genes that were significantly upregulated in the MPO high group. | | | | | |
| Control Signature: genes that were significantly downregulated in the MPO low group. | | | | | |

**Table S2. Mouse and human flow cytometric antibody panels.**

| Panel | Antibody | Dilution | Clone | Company | Catalog number |
| --- | --- | --- | --- | --- | --- |
| Tumor-infiltrating lymphoid immune cells  (mouse)  or  Immune populations of spleens (mouse) | CD45-AF700 | 1:200 | 30-F11 | BioLegend | 103128 |
|  | CD3-BUV395 | 1:40 | 145-2C11 | BD Biosciences | 563565 |
|  | CD8-PerCPCy5.5 | 1:80 | 53-6.7 | BioLegend | 100734 |
|  | CD4-BUV496 | 1:80 | GK1.5 | BD Biosciences | 564667 |
|  | γδTCR-PECF594 | 1:40 | GL3 | BD Biosciences | 563532 |
|  | PD-1-APC | 1:40 | 29F.1A12 | BioLegend | 135210 |
|  | CD62L-BV605 | 1:50 | MEL-14 | BioLegend | 104438 |
|  | CD44-BUV737 | 1:160 | IM7 | BD Biosciences | 612799 |
|  | NKp46-BV510 | 1:20 | 29A1.4 | BioLegend | 137623 |
|  | CD19-FITC | 1:160 | 6D5 | BioLegend | 115506 |
|  | CD25-BV785 | 1:80 | PC61 | BioLegend | 102051 |
|  | FoxP3-PE | 1:40 | FJK-16s | eBioscience ThermoFisher | 12-5773-82 |
| Tumor-infiltrating myeloid immune cells  (mouse)  or  Immune populations of spleens (mouse) | CD45-BV785 | 1:160 | 30-F11 | BioLegend | 103149 |
|  | Ly6C-APC | 1:79 | HK1.4 | BioLegend | 128015 |
|  | Ly6G-PE /Dazzle | 1:166 | 1A8 | BioLegend | 127648 |
|  | CD11c-BV605 | 1:20 | N418 | BioLegend | 117334 |
|  | PD-L1-PeCy7 | 1:79 | 10F.9G2 | BioLegend | 124313 |
|  | CD206-FITC | 1:160 | C068C2 | BioLegend | 141703 |
|  | MHCII-PerCP-Cy5.5 | 1:160 | M5/114.15.2 | BioLegend | 107625 |
|  | CD103-BV510 | 1:40 | 2E7 | BioLegend | 121423 |
|  | CD11b-BUV737 | 1:80 | M1/70 | BD Biosciences | 612801 |
|  | F4/80-BUV395 | 1:40 | T45-2342 | BD Biosciences | 565614 |
|  | Siglec-F-PE | 1:40 | E50-2440 | BD Biosciences | 562068 |
| Immune populations of thymus of nontumor-bearing mice | CD3-BUV395 | 1:40 | 145-2C11 | BD Biosciences | 563565 |
|  | CD8-PerCPCy5.5 | 1:80 | 53-6.7 | BioLegend | 100734 |
|  | CD4-BUV496 | 1:80 | GK1.5 | BD Biosciences | 564667 |
|  | CD25-BV785 | 1:80 | PC61 | BioLegend | 102051 |
|  | CD44-BUV737 | 1:160 | IM7 | BD Biosciences | 612799 |
| Interferon-gamma expression (mouse) | CD45-FITC | 1:100 | 30-F11 | BioLegend | 103108 |
|  | CD3-BV421 | 1:25 | 145-2C11 | BioLegend | 100336 |
|  | CD4-PE-Cy7 | 1:50 | RM4-5 | Biolegend | 100528 |
|  | CD8-PerCP-Cy5.5 | 1:80 | 53-6.7 | BioLegend | 100734 |
|  | IFN-γ-PE | 1:50 | XMG1.2 | BioLegend | 505808 |
| T cell proliferation  (human) | CD8a-FITC | 1:100 | RPA-T8 | BioLegend | 301006 |
| Interferon-gamma expression (human) | CD8-APC | 1:25 | RPA-T8 | BioLegend | 301014 |
|  | IFN-γ-BUV395 | 1:20 | B27 | BD Biosciences | 563563 |
| T cell cytotoxicity (human) | CD8-APC | 1:25 | RPA-T8 | BioLegend | 301014 |
|  | Granzyme-B-FITC | 1:25 | GB11 | BioLegend | 515403 |
|  | Perforin-PE | 1:25 | B-D48 | BioLegend | 353304 |
| MPO binding/internalization (human) | CD8-BV510 | 1:50 | RPA-T8 | BioLegend | 301006 |
|  | MPO-FITC | 1:2.5 | 5B8 | BD Biosciences | 333138 |
| MPO in lymphocytes in human NSCLC tumors | CD45-BV510 | 1:100 | H130 | BioLegend | 304035 |
|  | CD66b-APC | 1:50 | G10F5 | BioLegend | 305118 |
|  | CD163-BV605 | 1:25 | GH1/61 | BioLegend | 333616 |
|  | MPO-FITC | 1:2.5 | 5B8 | BD Biosciences | 333138 |
| Functional analysis of CD8^+^ T cells in human NSCLC tumors | CD45-BV510 | 1:300 | H130 | BioLegend | 304035 |
|  | CD3-AF700 | 1:25 | UCHT1 | BioLegend | 300424 |
|  | CD4-BUV395 | 1:40 | RPA-T4 | BD Biosciences | 563550 |
|  | CD8-BV605 | 1:50 | RPA-T8 | BioLegend | 301040 |
|  | MPO-FITC | 1:5 | 5B8 | BD Biosciences | 333138 |
|  | CD69-BV421 | 1:100 | FN50 | BioLegend | 310930 |
|  | PD1-BUV737 | 1:66 | EH12.1 | BD Biosciences | 612792 |

**Fig. S1**

**Validation of MPO signature.**

**(A)** Scatter plot showing Pearson correlation between MPO transcript and protein expression in the CPTAC LUAD dataset. **(B)** Scatter plot showing Pearson correlation between scaled MPO signature and scaled Neutrophil content in two different datasets (ICP3: Neutrophils in % CD45^+^ cells; TCGA-LUAD: Percentage of Neutrophils derived from CASSANDRA website). **(C)** Scatter plot showing Pearson correlation between scaled MPO signature and Control signature in ICP3 and TCGA LUAD dataset.

**Fig. S2**

**MPO content of KP and LLC tumors**

Analysis of KP **(A)** and LLC **(B)** tumors of WT and MPO-KO mice. Tumor homogenates were assessed using a mouse MPO ELISA. Data are presented as mean ± SD. Statistical differences were determined by unpaired Student’s *t*-tests (*****p* < 0.0001).

**Fig. S3**

**Flow cytometric gating strategies.**

Schematic representation of the flow cytometric gating strategies used to analyze single-cell suspensions from in vivo experiments and NSCLC tumors, and in vitro MPO-treated T cell experiments.

(A) **Tumor-infiltrating lymphoid immune cells (mouse) or immune populations of spleens (mouse).** T cells were gated as CD45^+^/CD3^+^; Natural Killer (NK) cells as CD45^+^/CD3^-^/NKp46^+^; B cells as CD45^+^/CD3^-^/CD19^+^; γδ T cells as CD45^+^/CD3^+^/γδTCR^+^; Natural Killer T (NKT) cells as CD45^+^/CD3^+^/NKp46^+^; CD8^+^ T cells as CD45^+^/CD3^+^/CD8^+^; CD4^+^ T cells as CD45^+^/CD3^+^/CD4^+^; CD8^+^ T effector (eff.) cells as CD45^+^/CD3^+^/CD8^+^/CD44^+^; CD8^+^ T naïve cells as CD45^+^/CD3^+^/CD8^+^/CD62L^+^; CD8^+^ T memory cells as CD45^+^/CD3^+^/CD8^+^/CD44^+^/CD62L^+^; CD4^+^ T effector (eff.) cells as CD45^+^/CD3^+^/CD4^+^/CD44^+^; CD4^+^ T naïve cells as CD45^+^/CD3^+^/CD4^+^/CD62L^+^; CD4^+^ T memory cells as CD45^+^/CD3^+^/CD4^+^/CD44^+^/CD62L^+^; and CD4^+^ T regulatory (T regs) as CD45^+^/CD3^+^/CD4^+^/FoxP3^+^. PD-1 expression was expressed as percentage of CD4^+^ and CD8^+^ T cells.
(B) **Tumor-infiltrating myeloid immune cells (mouse) or immune populations of spleens (mouse).**  Eosinophils were gated as CD45^+^/CD11b^+^/CD11c^-^/Siglec-F^+^; neutrophils as CD45^+^/CD11b^+^/CD11c^-^/Siglec-F^-^/Ly6G^+^; monocytes as CD45^+^/CD11b^+^/CD11c^-^/Siglec-F^-^/Ly6G^-^/Ly6C^+^ and macrophages as CD45^+^/CD11b^+^/CD11c^-/+^/Siglec-F^-^/Ly6G^-^/Ly6C^-^/F4/80^+^; myeloid dendritic cells (mDCs) as CD45^+^/CD11b^+^/ F4/80^+/-^/CD11c^+^/MHCII^+^; plasmacytoid DCs (panDCs) as CD45^+^/CD11b^-^/ F4/80^-^/MHCII^+^ and dendritic cells (DCs2) CD45^+^/CD11b^-^/ F4/80^-^/MHCII^+^/CD103^+^.
(C) **Immune populations of thymus of nontumor-bearing mice.** T cells were gated as CD45^+^/CD3^+^; CD8^+^ T cells as CD45^+^/CD3^+^/CD8^+^; CD4^+^ T cells as CD45^+^/CD3^+^/CD4^+^; CD4^-^/CD8^-^ double negative (DN) T cells were further gated on differentiation stage DN1-DN4 via CD44 and CD25 expression.
(D) **MPO in tumors from patients with NSCLC**. CD66b marker was used to exclude myeloid cells within the lymphoid gate and MPO presence in lymphocytes was determined as CD66b^-^/MPO^+^. MPO^+^ neutrophils were gated as CD163^-^/CD66b^+^/MPO^+^.
(E) **MPO uptake of MPO-treated human CD8^+^ T cells from healthy blood donors**. MPO^+^ CD8^+^ T cells were gated as CD8^+^/CD4^-^/MPO^+^.
(F) **Functional analysis of MPO^+/-^ CD8^+^ T cells in tumors from patients with NSCLC.** CD8^+^ T cells were gated as CD45^+^/CD3^+^/CD8^+^. MFI of CD69 and PD-1 was shown in MPO^+^ and MPO^-^ CD8^+^ T cells.

**Fig. S4**

**Infiltrating immune cells in LLC tumors.**

**(A–H)** Flow cytometric analysis of single-cell suspensions of LLC tumors from MPO wildtype (WT) and knockout (KO) mice. Data are presented as mean ± SD (n = 15-16). Statistical differences were determined using unpaired Student’s *t*-tests (**p* < 0.05, ***p* < 0.01, ****p* < 0.001 and *****p* < 0.0001).

**Fig. S5**

**Splenic controls of tumor-free mice.**

**(A-E)** Flow cytometric analysis showing immune cell content of spleens from tumor-free mice. Data are presented as mean ± SD (n = 6-10). Statistical differences were determined using unpaired Student’s *t*-tests. No significant differences were found.

**Fig. S6**

**Thymic controls of tumor-free mice.**

**(A-E)** Flow cytometric analysis showing immune cell content of thymus from tumor-free mice. Data are presented as mean ± SD (n = 7-10). Statistical differences were determined using unpaired Student’s *t*-test (**p* < 0.05 and ***p* < 0.01).

**Fig. S7**

**Splenic controls of KP tumor-bearing mice.**

**(A-E)** Flow cytometric analysis showing immune cell content on spleens from tumor-bearing mice. Data are presented as means ± SD (n = 7). Statistical differences were determined using unpaired Student’s *t*-test. No significant differences were found.

**Fig. S8**

**CD8^+^ T cells depletion controls.**

**(A)** Anti-CD8 antibody or isotype control was intraperitoneally (i.p.) administered one day prior to subcutaneous (s.c.) engraftment of 5×10^5^ KP cells into MPO^+/+^ (WT) and MPO^-/-^ (KO) mice. 500 µg and 250 µg of anti-CD8 antibody or isotype were administered on days 6 and 11, respectively. Blood was drawn on days 0, 5 and 11. **(B)** Expression of CD8^+^ T cells was determined by flow cytometry. Data are presented as mean ± SD (n = 3). Statistical differences were determined using unpaired Student’s *t*-test (**p<0.01, ***p<.001).

**Fig. S9**

**Immune cell infiltration in CD8^+^ depleted tumors of wildtype and MPO-knockout mice.**

**(A-E)** Flow cytometric analysis showing immune cell content in tumors depleted (dep) of CD8^+^ T cells and isotype control (iso) of WT and MPO-KO mice. Data are presented as mean ± SD (n = 5-9). Statistical differences were determined using ordinary one-way ANOVA with Tukey’s multiple comparisons test (*p<0.05, **p<0.01, ***p<.001).
